# The Role of Breast Examination in Prenatal Care: A Case Report

**DOI:** 10.7759/cureus.94008

**Published:** 2025-10-07

**Authors:** Anastasia Mavridou, Konstantinos Samartzis, Evaggelos Alexopoulos, Sotirios Kalogeropoulos

**Affiliations:** 1 Department of Obstetrics and Gynecology, Alexandra Hospital, Athens, GRC

**Keywords:** antenatal screening, breast examination, breast tumors, pregnancy-associated breast cancer, prenatal care

## Abstract

Pregnancy-associated breast cancer (PABC) is a relatively rare but clinically significant condition. Diagnosis is often delayed, as physiological breast changes during pregnancy can obscure malignant findings, resulting in more advanced disease at presentation. PABC poses unique diagnostic and therapeutic challenges due to its rarity and the complexity of managing maternal and fetal health. This case highlights these challenges in a 35-year-old woman at 30 weeks of gestation who presented with a large, painful left breast mass. Open biopsy confirmed grade 3, triple-negative, infiltrating ductal carcinoma. Axillary lymphadenopathy was present, and staging revealed liver, bone, and lung metastases. Neoadjuvant chemotherapy was administered, followed two weeks after the last dose by cesarean delivery at 37+5 weeks, resulting in the birth of a healthy infant without complications. Despite the initial response to treatment, the disease progressed three months later. Molecular therapy was subsequently administered, but the patient ultimately succumbed 16 months after the initial diagnosis. Current guidelines do not recommend routine breast examinations during antenatal care; however, this case underscores the importance of breast evaluation during pregnancy as both a medical and psychological imperative.

## Introduction

Pregnancy-associated breast cancer (PABC), defined as breast cancer diagnosed during pregnancy or within the first postpartum year [1], poses unique clinical challenges due to its interaction with pregnancy-related hormonal changes and typically aggressive tumor biology [2]. Although PABC is rare, accounting for 3-7% of breast cancers in women under 40 years old [3], its incidence is rising due to delayed childbearing and increasing breast cancer rates [4]. PABC often demonstrates aggressive behavior compared with breast cancer in non-pregnant women of similar age, with higher rates of triple-negative phenotype, lymph node involvement, and distant metastases [5,6]. Its management is complicated by the dual challenge of optimizing maternal treatment while safeguarding fetal health. Current clinical guidance for breast examination during pregnancy remains limited, particularly for women without recognized high-risk factors, and standardized screening recommendations are lacking [7]. This case report highlights these unique challenges through a case encountered in our department.

## Case presentation

**Figure 1:**
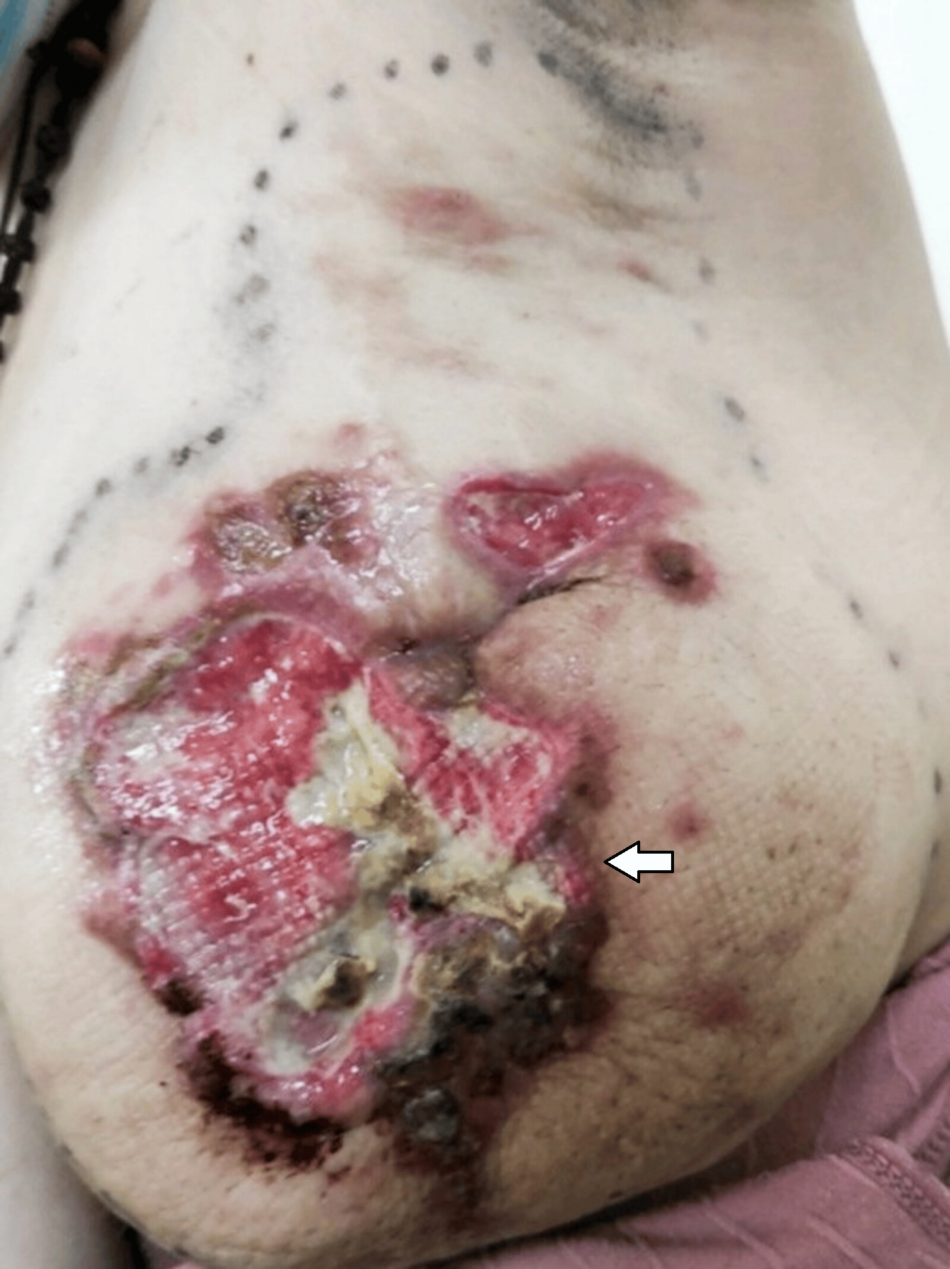
The patient presented at 30 weeks of gestation.

A 35-year-old woman at 30 weeks of gestation presented with a four-month history of a large, painful left breast mass. She had a medical history of epilepsy but no family history of breast cancer. On physical examination, a large lateral left breast mass was noted, associated with overlying warmth, thickened skin with ulcerated lesions, and palpable left axillary lymphadenopathy (Figure 1).

**Figure 2:**
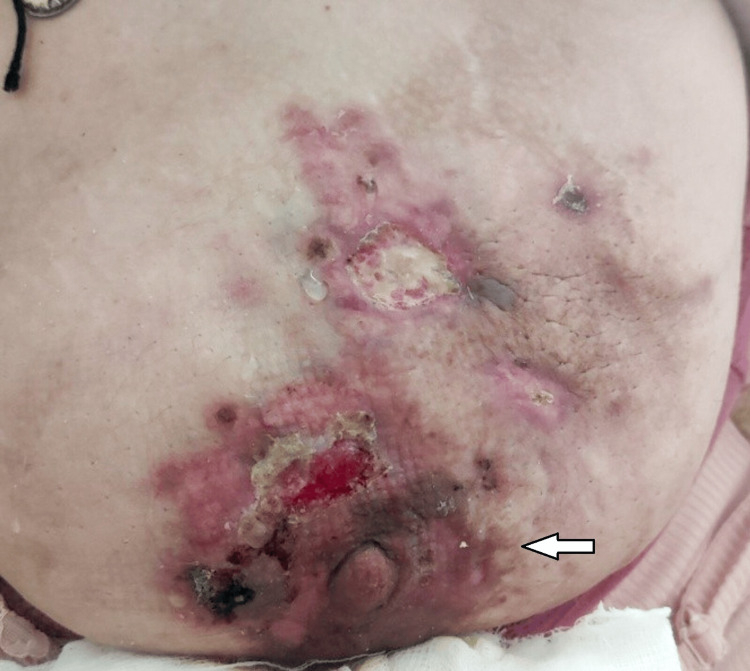
The patient at 35 weeks of gestation, right after administration of neoadjuvant chemotherapy, resulting in significant disease control and clinical improvement.

The patient reported severe pain, limiting cooperation. The diagnostic approach aimed to distinguish malignant from benign pregnancy-related breast conditions. Two prior cytology assessments, performed four and two months earlier, were negative, prompting an open biopsy for definitive diagnosis. Ultrasound and MRI were selected to assess lesion extent and potential metastases while minimizing fetal exposure. Alternative causes, such as mastitis or galactocele, were considered but excluded based on the persistent mass, skin changes, axillary lymphadenopathy, and lack of response to conservative management. An open biopsy confirmed triple-negative (ER 0%, PR 0%, HER2 negative, Ki 67>50%), grade 3 infiltrating ductal carcinoma extending into the skin, and staging with ultrasound, MRI, and laboratory testing demonstrated liver, bone, and lung metastases. A multidisciplinary team, including oncologists and obstetricians, recommended neoadjuvant chemotherapy. Weekly carboplatin (1.5 AUC) and paclitaxel (80 mg/m²) were administered for five cycles until 35 weeks of gestation, resulting in significant disease control and clinical improvement (Figure 2).

**Figure 3:**
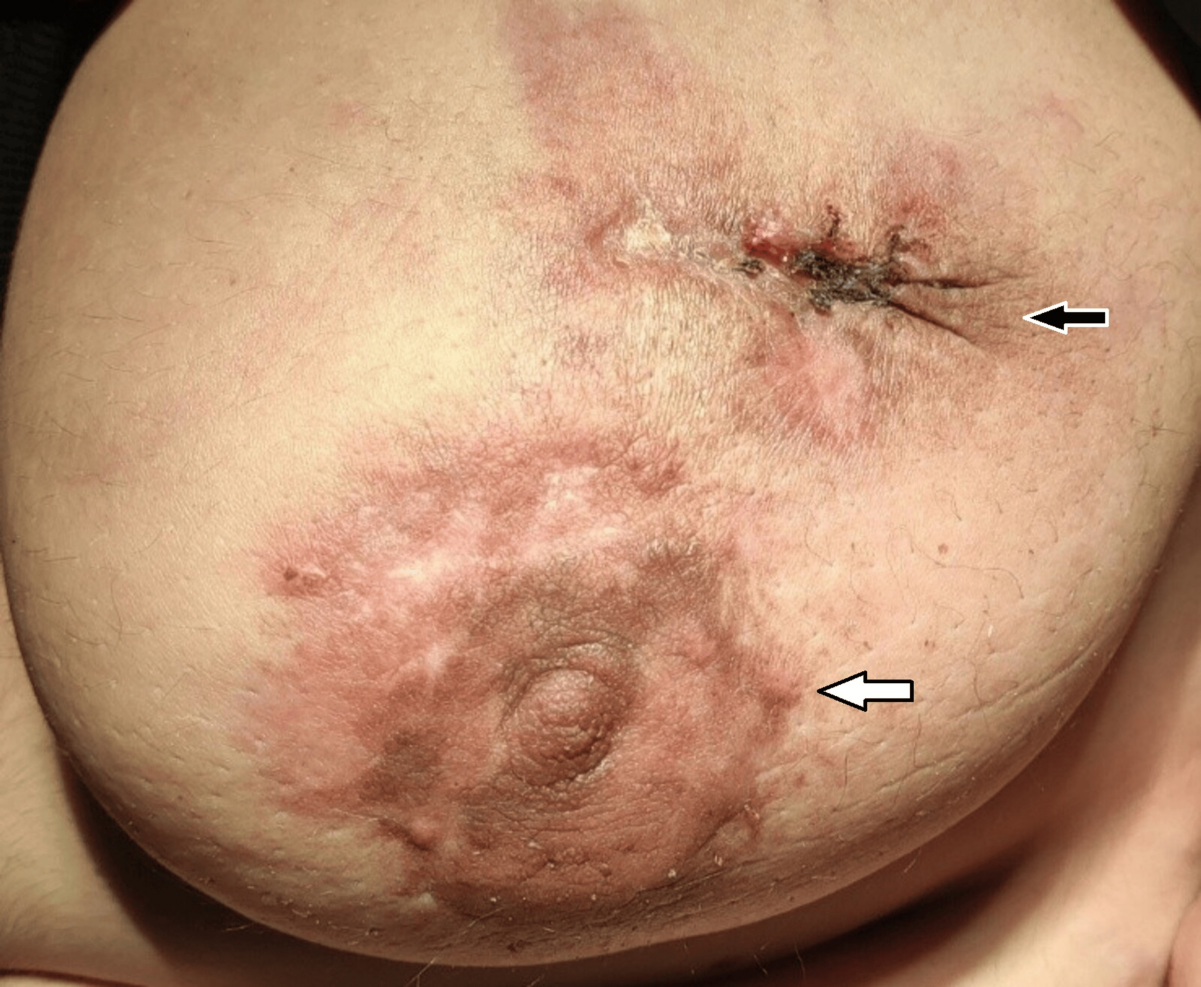
After neoadjuvant chemotherapy and cesarean section. White arrow: Initial response to treatment Black arrow: Open biopsy site

**Figure 4:**
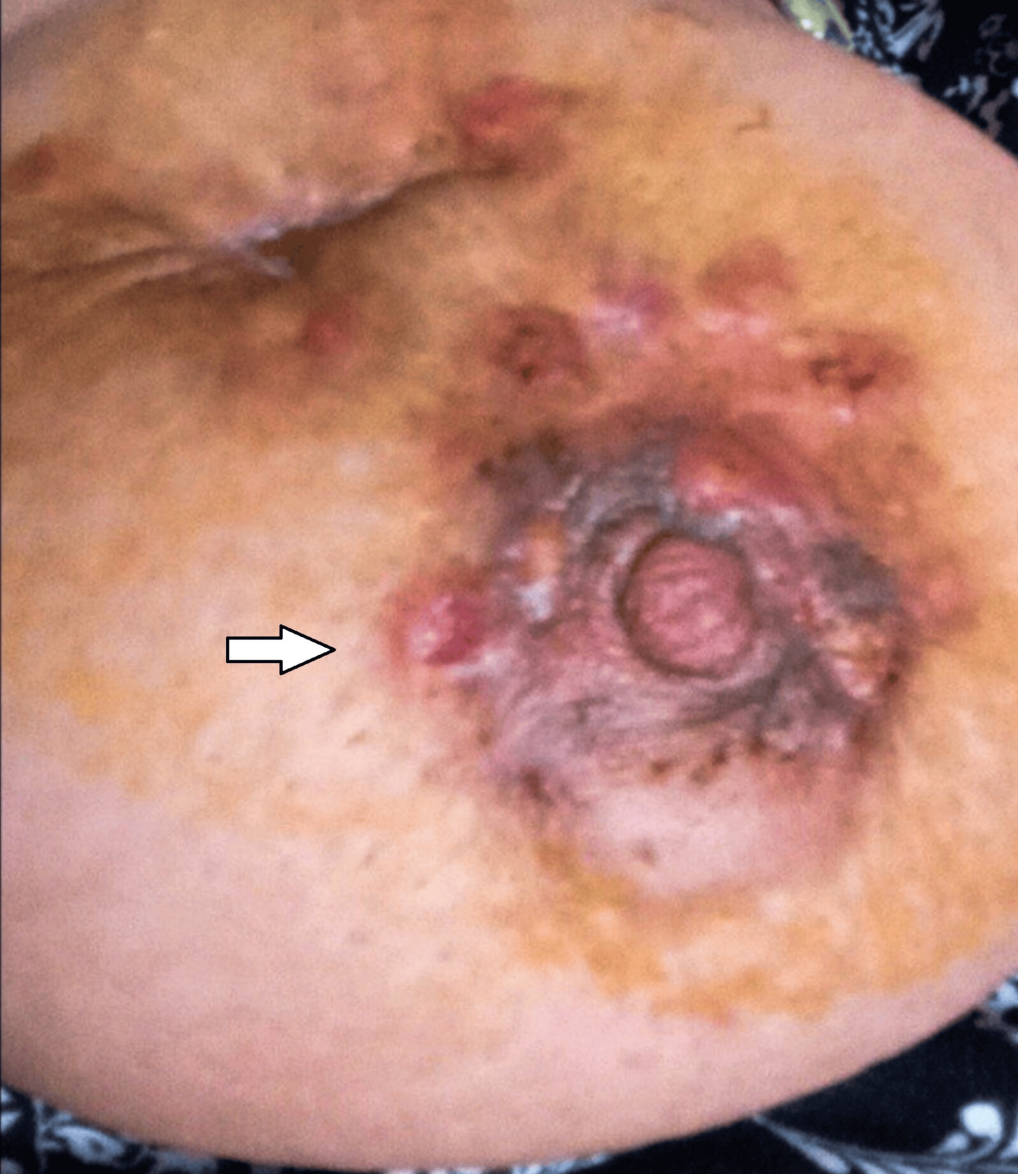
After neoadjuvant chemotherapy and cesarean section.

At 37+5 weeks, two weeks after the last chemotherapy dose, cesarean delivery was performed. A healthy female infant weighing 2590 g was delivered without complications. Neonatal evaluation was normal. Postpartum staging confirmed persistent lung and liver metastases and secondary lesions in the sternum and L3 vertebra. BRCA gene testing revealed no mutations. Chemotherapy was resumed seven days postpartum (Figures 3, 4).

**Figure 5:**
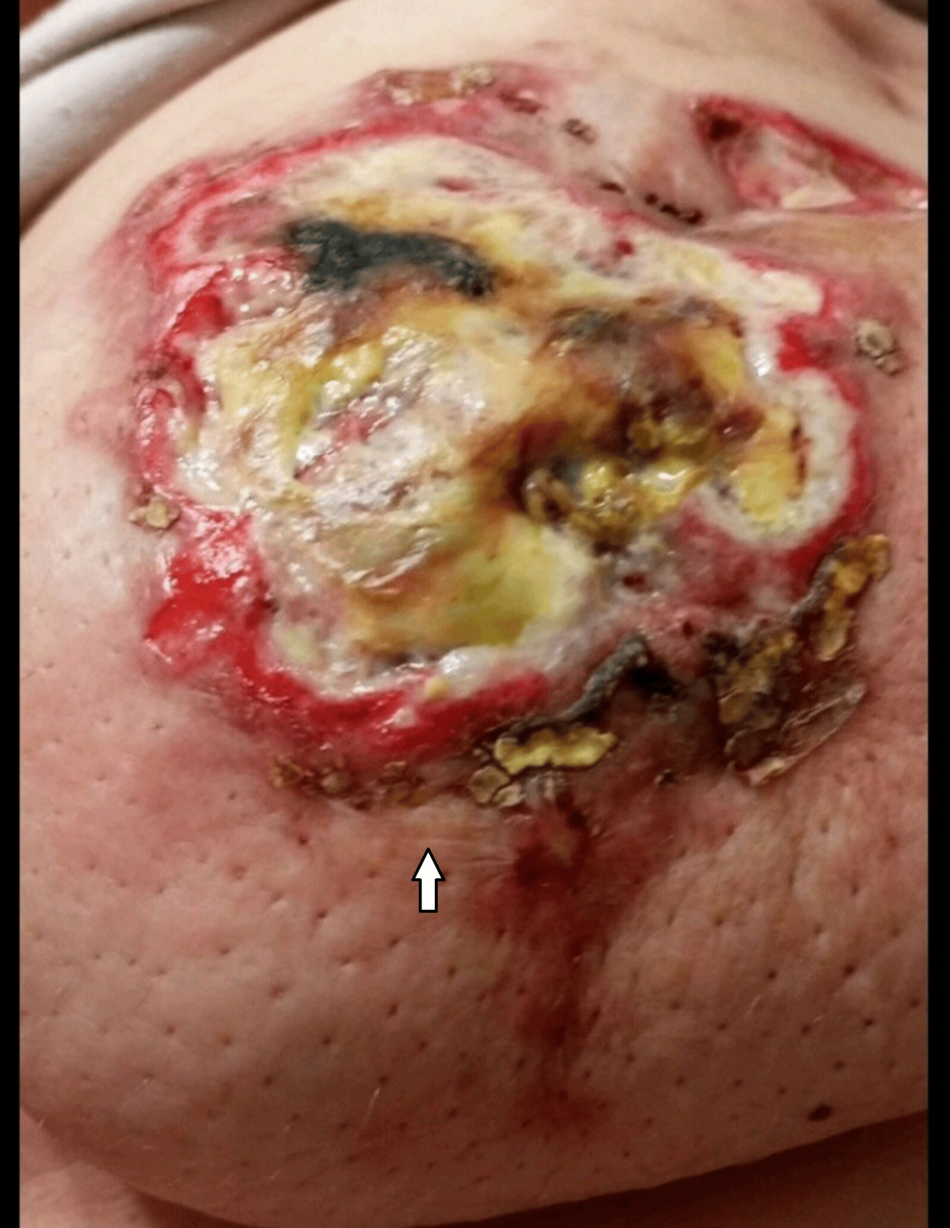
Despite the initial treatment response, the disease progressed.

Despite the initial response to treatment, the disease progressed three months later (Figure 5).

**Figure 6:**
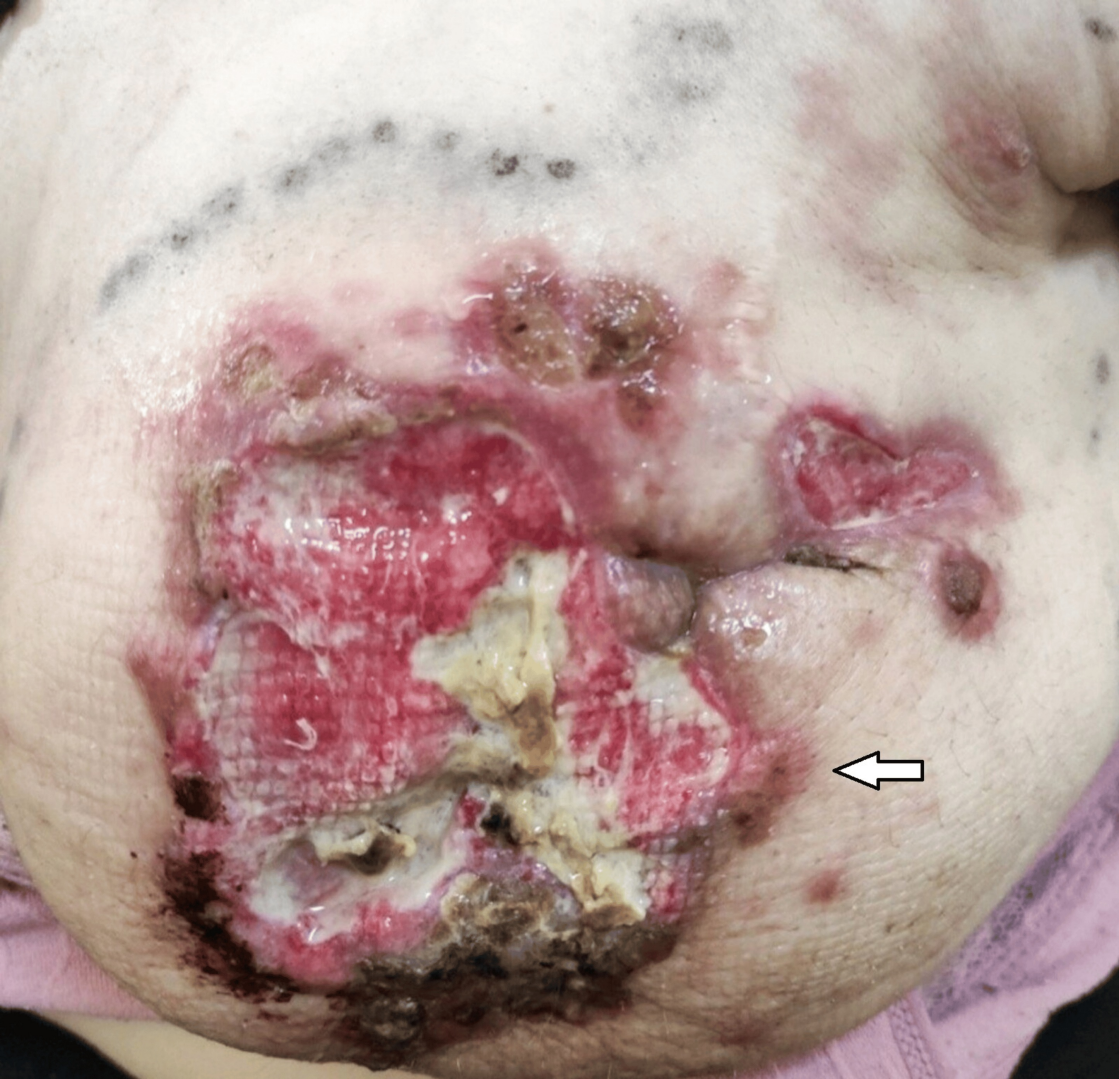
Due to an extended breast skin lesion, a comforting mastectomy was not indicated.

Due to extensive breast skin involvement, a palliative mastectomy was not indicated (Figure 6).

The patient received molecular therapy (sacituzumab govitecan-hziy) but ultimately succumbed 16 months after the initial diagnosis.

## Discussion

Prenatal care is essential for maternal and fetal health, encompassing assessments that ensure maternal well-being and optimal fetal development. Breast examination, though often overlooked, is a critical component of prenatal care. Early recognition of suspicious findings such as palpable masses, skin changes, or lymphadenopathy can facilitate timely diagnosis and intervention. However, pregnancy-related physiological changes may obscure pathological findings, leading to diagnostic delays and more advanced disease at presentation.

In this case, the patient first attended the outpatient clinic in late pregnancy. Current guidelines generally do not recommend routine breast examinations during antenatal care. HSOG considers it non-mandatory; NCCN does not advise screening for women under 40 without high-risk features. RCOG, Cochrane, ACOG, WHO, NICE, ASCO, ESMO, and St. Gallen provide limited guidance on this topic [8].

The diagnosis of PABC remains particularly challenging, as the profound physiological changes of the breast during pregnancy and lactation, such as engorgement, increased density, and nodularity, can mask, mimic, or delay the recognition of malignant lesions. These alterations often lead to misattribution of symptoms, such as pain, palpable masses, or skin changes, to benign pregnancy-related processes. Furthermore, the pathophysiology of PABC reflects a complex interplay between the unique hormonal and immunological milieu of pregnancy and the aggressive biological behavior of breast cancer, contributing to its frequently advanced stage at presentation and poorer outcomes compared to age-matched non-pregnant patients [5,9]. Imaging modalities such as ultrasound and mammography are valuable for lesion detection, while fine-needle aspiration and core needle biopsy provide definitive histopathological confirmation [8,10].

Optimal management of PABC requires a multidisciplinary approach with individualized treatment planning based on tumor biology, gestational age, and patient preferences, while simultaneously considering both maternal and fetal health [4]. Surgery remains the cornerstone of therapy, with breast-conserving approaches feasible in selected cases. Chemotherapy can be administered during the second and third trimesters, after organogenesis. Hormonal and targeted therapies (i.e., immunotherapy) are limited due to potential teratogenicity and limited safety data during pregnancy [4]. While this report provides valuable insights, it is inherently limited by its single-patient nature, which prevents generalization and may be influenced by reporting bias. Additionally, long-term maternal and infant follow-up data were not available, restricting conclusions regarding outcomes beyond the early postpartum period. Nevertheless, despite these limitations, the case highlights clinically relevant issues and underscores the importance of heightened awareness and vigilance for PABC during routine prenatal care.

## Conclusions

PABC presents unique diagnostic and management challenges due to its rarity and the need to balance maternal therapy with fetal safety. This case underscores the critical importance of vigilant clinical evaluation and targeted imaging during pregnancy for early diagnosis among gynecologists and breast disease specialists. While routine breast examination for all pregnant women cannot be recommended based on a single case, this report highlights the need for heightened awareness and consideration of breast evaluation as both a medical and psychological imperative. Furthermore, tailored screening programs are essential, as PABC patients often lack specific risk factors that would otherwise prompt earlier or more intensive surveillance.
